# The Ras/MAPK Pathway Is Required for Generation of iNKT Cells

**DOI:** 10.1371/journal.pone.0019890

**Published:** 2011-05-10

**Authors:** Taishan Hu, Idoia Gimferrer, Amie Simmons, David Wiest, José Alberola-Ila

**Affiliations:** 1 Immunobiology and Cancer Research Program, Oklahoma Medical Research Foundation, Oklahoma City, Oklahoma, United States of America; 2 Immune Cell Development and Host Defense Program, Fox Chase Cancer Center, Philadephia, Pennsylvania, United States of America; 3 Department of Cell Biology, University of Oklahoma Health Sciences Center, Oklahoma City, Oklahoma, United States of America; Centre de Recherche Public de la Santé (CRP-Santé), Luxembourg

## Abstract

iNKT cells derive from CD4^+^CD8^+^ DP thymocytes, and are selected by thymocyte-thymocyte interactions through signals from their invariant Vα14-Jα18 TCR and from the costimulatory molecules SLAMF1 and SLAMF6. Genetic studies have demonstrated the contribution of different signaling pathways to this process. Surprisingly, current models imply that the Ras/MAPK pathway, one of the critical mediators of conventional αβ T cell positive selection, is not necessary for iNKT cell development. Using mice defective at different levels of this pathway our results refute this paradigm, and demonstrate that Ras, and its downstream effectors Egr-1 and Egr-2 are required for positive selection of iNKT cells. Interestingly our results also show that there are differences in the contributions of several of these molecules to the development of iNKT and conventional αβ T cells.

## Introduction

Invariant NKT (iNKT) cells are a subset of αβ T cells characterized by the expression of an invariant Vα14-Jα18 TCR (Vα24-Jα18 in humans). iNKT cells recognize lipid antigens presented by the non-polymorphic, MHC I-like molecule, CD1d [1]. iNKT have been implicated in multiple processes, including microbial immunity, tumor rejection, autoimmunity, atherosclerosis and allergy [2], [3].

iNKT cells develop in the thymus from CD4^+^CD8^+^ double positive cells [2], [4]-[6] and require the cooperative engagement of the TCR and members of the signaling lymphocytic–activation molecule (SLAM) family expressed on DP thymocytes [7]. Some of the signaling pathways involved in iNKT development downstream of the TCR have been identified, including itk kinases [8], NFκB [9], [10], and Calcineurin [11].

The Ras/MAPK pathway plays a central role during αβ T cells positive selection [12], [13] but it is thought to be dispensable for iNKT cell development [14]–[16]. In fact, it is proposed that one of the roles of SLAMs in positive selection of iNKT cells is to block activation of Ras, by inducing recruitment and activation of Ras-GAP [14], [15].

In this manuscript we present genetic evidence supporting a critical role of Ras, and its downstream effectors Egr-1, Egr-2 for positive selection of iNKT cells, suggesting that the signaling pathways emanating from the TCR during positive selection of conventional αβ T cells and iNKT cells are similar.

## Results

### Generation of iNKT cells is blocked in dnRas transgenic mice

The Ras/MAPK pathway plays a central role in the control of positive selection of conventional αβ T cells [12], [13], [17], but for a long time, and based on one early report [16] it has been assumed that it was not necessary for the generation of iNKT cells -see [14], [15]-. Since in the original paper NKT cell development had only been assessed using NK1.1 staining in the thymus, we decided to revisit the possible contribution of Ras to iNKT cell development using CD1d-tetramers.

**Figure 1:**
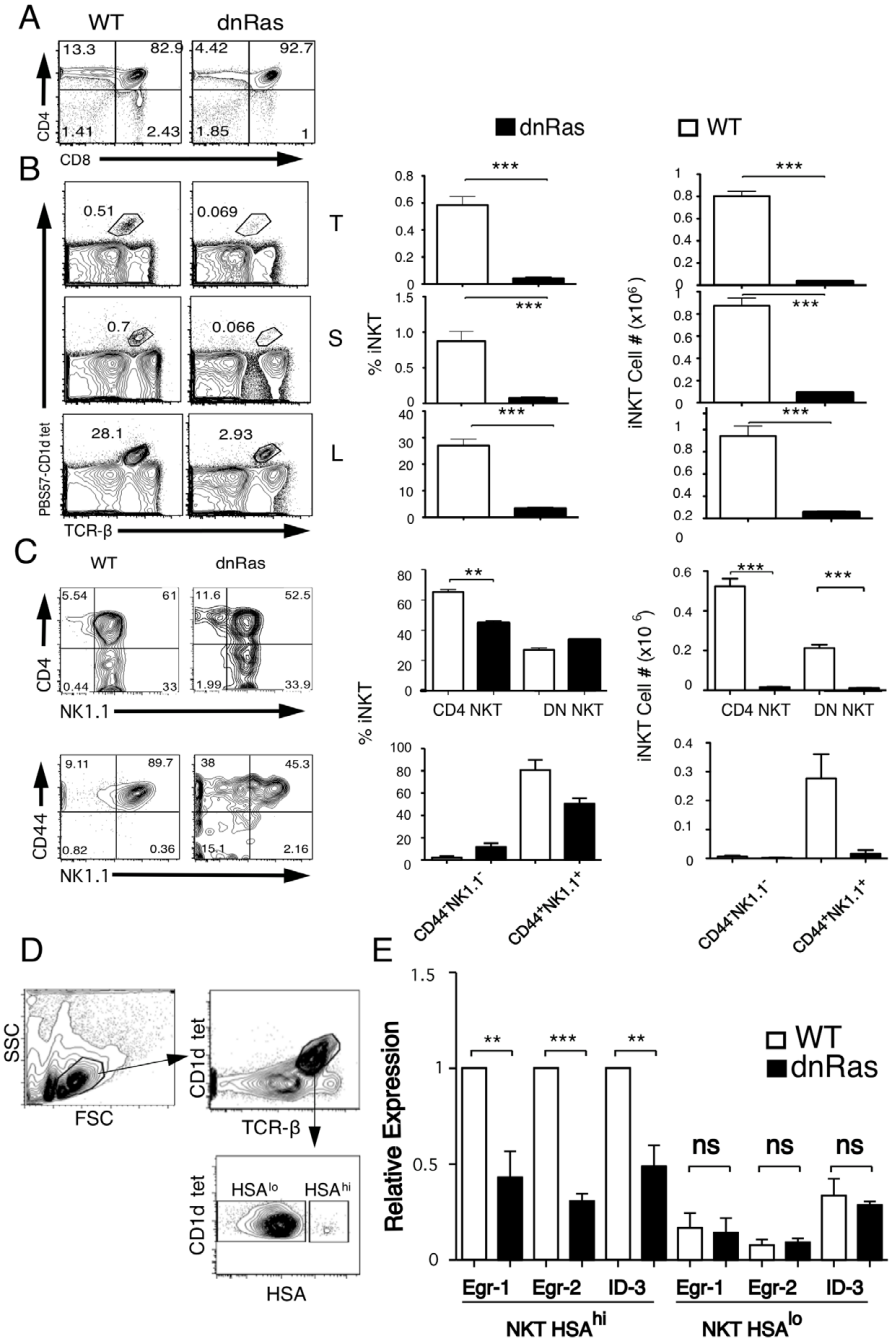
dnRas mice lack iNKT cells. (**a**) Thymic profile of WT and dnRas mice (**b**) Percentages and absolute numbers of iNKT cells in the thymus (T), spleen (S) and liver mononuclear cells (L) of normal littermate controls (WT) and dnRas mice stained with CD4, CD8, PBS57-loaded CD1d tetramer and TCRβ. (**c**) Percentages and absolute numbers of iNKT cell subpopulations in gated TCR^hi^PBS57-CD1dtet^+^ thymocytes from WT and dnRas mice. Results representative of nine independent dnRas and WT pairs analyzed in five experiments**,** except for the CD44/NK1.1 histograms (n = 2). The bar graphs show the average and SEM of all the experiments. Significance as assessed using a two-tailed unpaired t-test. ***<0.001, **<0.01. (**d**) Gating strategy used to sort the different populations. (**e**) Expression of Egr-1, Egr-2 and Id3 in sorted Tet^+^ HSA^hi^ and Tet^+^ HSA^lo^ from normal littermate control (WT) and dnRas mice. Bar graphs show relative expression of dnRas compared to WT for three independent experiments. Each experiment was an independent sort of a WT and dnRas pair. Expression in each experiment was normalized to the expression levelis in Tet^+^ HSA^hi^ WT cells. Significance as assessed using a two-tailed unpaired t-test. **<0.01.

We analyzed transgenic mice expressing a dominant negative Ras, RasN17 [12], using PBS57-loaded CD1d tetramers to quantify iNKT cells in the thymus, liver and spleen. As shown in Fig. 1. we found a dramatic (∼90%) decrease in the percentage and total numbers of iNKT cells present in dnRas transgenic mice, suggesting that, despite previous reports and the general consensus in the field, Ras plays a central role in regulating positive selection of iNKT cells. The few iNKT cells present in the thymus of dnRas mice accumulate in the early immature populations (CD44^-^NK1.1^-^ and CD44^+^NK1.1^-^) (Fig 1C) where they are present at almost normal numbers (and increased percentages), suggesting that the block in iNKT cell development imposed by dnRas is at early stages of positive selection, similar to its function during conventional αβ T cell development.

During development of conventional αβ T cells Ras has been placed upstream of the transcription factors Egr and Id3 [13], [18]. To test whether expression of these transcription factors was also altered in iNKT cell precursors we sorted CD1d tetramer positive, HSA^hi^ (immature) and HSA^lo^ (mature) cells from wild type (WT) and dnRas mice, and analyzed the expression of these transcription factors in the different subsets. As shown in Fig. 1D, dnRas thymocytes have decreased levels of Egr-1, Egr-2 and Id3, suggesting that a similar signaling cascade is implicated in the development of iNKT cells.

### Role of Egr1 and Egr2 in the generation of iNKT cells

The Egr family of transcription factors plays an important role in many developmental checkpoints during T cell development. A recent series of experiments showed that Egr-2 plays an important role in iNKT development, while Egr-1 was apparently dispensable [11]. Given our results with dnRas, we decided to revisit these experiments.

**Figure 2:**
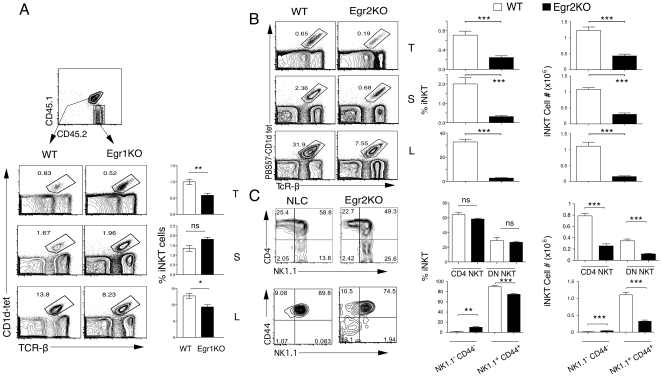
Egr1 and Egr2 contribute in a quantitatively different manner to iNKT cell development. (**a**) Contribution of WT and Egr1^-/-^ (Egr1KO) cells to the iNKT compartment in thymus (T), spleen (S) and liver (L) of mixed bone marrow chimeras generated by injecting Egr1^-/-^ (CD45.2) and F1(C57BL/6xB6-LY5.2/Cr) (CD45.1;CD45.2) bone marrow cells into lethally irradiated B6-LY5.2/Cr recipient mice (CD45.1). Mean and SEM is shown on the right (n = 5). Significance was assessed using a paired t-test. **<0.01, *<0.05. This is one out of two independent experiments. **(b)** Percentages and absolute numbers of iNKT cells in the thymus (T), spleen (S) and liver mononuclear cells (L) of WT and Egr-2^f/f^-*lck*-Cre (EGR2KO) mice stained with CD4, CD8, PBS57-loaded CD1d tetramer and TCRβ. **(c)** Percentages and absolute numbers of iNKT cell subpopulations in gated TCR-β^hi^PBS57-CD1dtet^+^ thymocytes from WT and EGR2KO mice. Results representative of five independent pairs in three independent experiments. The bar graphs show the average and SEM of all the experiments. Significance was assessed using an unpaired t-test. ***<0.001, **<0.01.

First we analyzed iNKT cell development in Egr-1 knockout mice. As described [11], the percentages and total numbers of iNKT cells in the thymus, spleen and liver of these animals do not seem affected (data not shown). However, because subtle defects may not be apparent in a non-competitive situation, we decided to test the ability of Egr-1^-/-^ thymocytes to give rise to iNKT cells in a competitive bone marrow chimera. Lethally irradiated CD45.1 hosts were reconstituted by a 1∶1 mix of Lin^-^ bone marrow cells from Egr-1^-/-^ (CD45.2) and CD45.1/CD45.2 F1 C57B/6 mice. After 8-12 weeks the chimeras were analyzed by flow cytometry to determine whether Egr-1^-/-^ DP thymocytes could develop into iNKT cells in the presence of WT DP. Our results (Fig. 2A) show that Egr-1^-/-^ thymocytes are significantly less able to contribute to the iNKT lineage than WT thymocytes, suggesting that the lack of phenotype in the Egr-1 knockout reflects compensation by other family members, most likely Egr-2. A similar result was observed in the liver population, while in the spleen the contribution of WT and Egr1^-/-^ was not significantly different.

It was recently shown that Egr-2^-/-^ fetal liver cells are defective in the generation of iNKT cells in fetal liver chimeras [11]. To confirm these results we used Egr-2^f/f^-*lck*-Cre mice [19], [20]. In these mice we observed a significant (∼50%) decrease in the percentages and numbers of iNKT cells in thymus, liver and spleen (Fig. 2B). As in the dnRas mice, there was an increase in the percentages of the more immature population (CD44^-^NK1.1^-^) (Fig. 2C). This defect is less profound that the defect reported in the fetal liver chimeras [11], suggesting that there could be differences between fetal and adult hematopoiesis in the contribution of different Egr family members to the generations of iNKT cells. We also performed competitive mixed bone marrow chimeras, and under these conditions Egr-2^-/-^ thymocytes have very profound defects in the generation of iNKT cells (data not shown).

**Figure 3:**
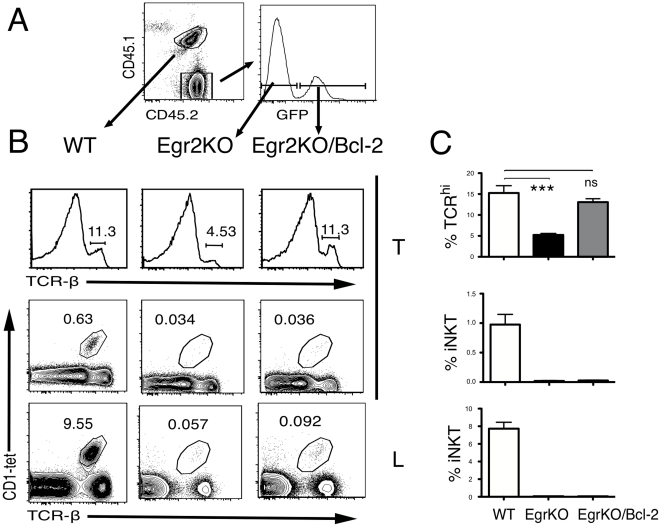
Bcl-2 can not rescue the iNKT defect in Egr2KOmice. **(a)** Donor contributions in bone marrow chimeras generated by injecting MiG-Bcl2 transduced Egr2^f/f^ *lck*-Cre (Egr2KO) (CD45.2) and wild-type (CD45.1;CD45.2) cells into lethally irradiated CD45.1 recipients, analyzed at 8–12 weeks (left), and a representative histogram of GFP expression in MiG-Bcl2 transduced Egr2KO thymocytes (right). **(b)** TcR thymic profiles (upper panel) and iNKT cells frequency (lower panels) in wild-type, Egr2KO and Egr2KO-Bcl2 donor cells in the thymus (T) and Liver (L) of chimeras described in (a). **(c)** Percentages of different cell subpopulations from WT Egr2KO and Egr2KO;Bcl-2 in the mixed bone marrow chimeras mice. Results representative of eight independent chimeras in two independent experiments. The bar graphs show the average and SEM of all the experiments. Significance as assessed using the unpaired t-test. ***<0.001.

These results suggested that Egr-2 plays a more central role that Egr-1 during the generation of iNKT cells. Since we have previously shown that overexpression of Bcl-2 can rescue the defect in positive selection of conventional αβ T cells observed in Egr2^f/f^-*lck*Cre mice [20], we decided to test whether Bcl-2 could also rescue generation of iNKT cells. We infected bone marrow Lin^-^ Sca^+^c-Kit^+^ Egr2^f/f^-cre cells with a retrovirus encoding for a Bcl2-GFP fusion protein [21], and adoptively transferred the infected cells into lethally irradiated Ly5.1 recipients. The mice were analyzed after 8 weeks, and the effect of Bcl-2 expression on the development of T cells and iNKT was assessed by comparing the percentages of TcR^high^ or CD1-tet^+^ thymocytes in GFP^+^ Egr2^f/f^-Cre vs. GFP^-^ Egr2^f/f^ cells. As shown in Fig. 3, overexpression of Bcl-2 increased the percentage of Egr2^f/f^-Cre TcR^hi^ single positive cells, as previously described [20], but could not rescue the defect in Egr2^f/f^-Cre iNKT cells. These experiments show that Bcl-2 is not sufficient to rescue development of the iNKT cell lineage in mice with genetic defects in Egr-2, although they do not discard its possible involvement. Furthermore, they demonstrate that the signaling requirements for the development of conventional αβ T cells and iNKT cells are different.

**Figure 4:**
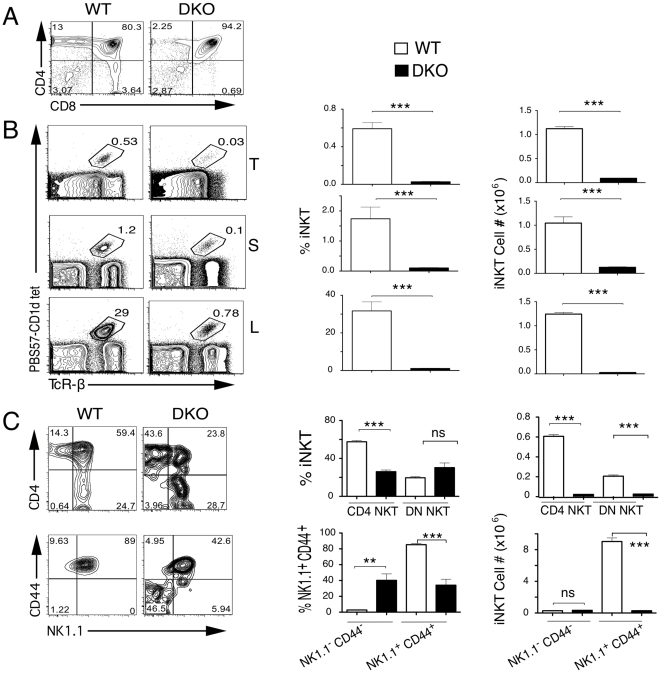
Egr1^-/-^; Egr2^f/f^ *lck*cre mice have a complete block in iNKT development. (**a**) Thymic profile of littermate wild-type (WT), or Egr1^-/-^;Egr2^f/f^ *lck*cre (DKO) mice **(b)** Percentages and absolute numbers of iNKT cells in the thymus (T), spleen (S) and liver mononuclear cells (L) of WT and EgrDKO mice stained with CD4, CD8, PBS57-loaded CD1d tetramer and TCRβ. **(c)** Percentages and absolute numbers of iNKT cell subpopulations in gated thymic TCR-βhiPBS57-CD1dtet^+^ of WT and EgrDKO mice. Results representative of five independent pairs in three independent experiments. The bar graphs show the average and SEM of all the experiments. Significance as assessed using the unpaired t-test. ***<0.001, **<0.01

To test whether the incomplete phenotype observed in the Egr-2^f/f^/*lck-*Cre mice was due to compensation by the related transcription factor Egr-1, we bred Egr-2^f/f^/*lck-*Cre mice to to Egr-1^-/-^ mice, and analyzed the phenotype of the Egr-1, Egr-2 double knockouts (DKO). As shown in Fig. 4, DKO mice have a very profound defect in the number and percentages of iNKT cells in thymus, spleen and liver, similar to that observed in dnRas mice. Similarly to dnRas mice, there was an increase in the percentages of the more immature population (CD44^-^NK1.1^-^) (Fig. 4C). These results suggest that both Egr-1 and Egr-2 play a role in iNKT cell development downstream Ras and calcineurin, although Egr-2 plays a more important role here than Egr-1, in contrast with their similar contributions to positive selection of conventional αβ T cells.

### dnRas thymocytes, but not Egr-1,2 DKO thymocytes, have small defects in SLAM family members and CD1d expression

**Figure 5:**
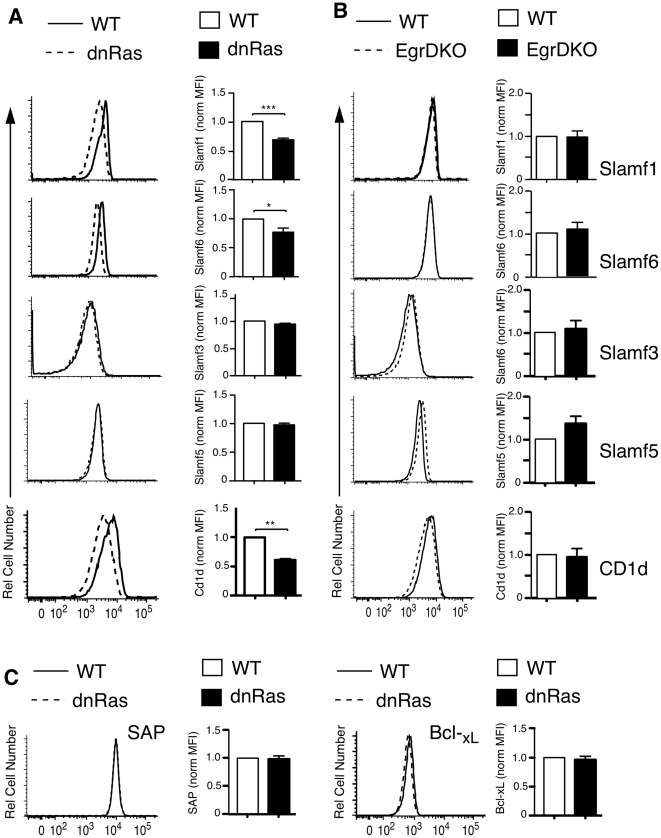
Defects in Slamf1, Slamf6 and CD1d expression in dnRas, but not Egr-1,2 double knockout mice. Slamf1, Slamf3, Slamf6, Slamf5 and CD1d expression levels in DP thymocytes mice were assessed by flow cytometry. Shown are representative histograms, and the mean and SEM of the normalized MFI of DP populations. In **(A)** WT vs. dnRas (n = 5). In **(B)** WT vs. Egr1^-/-^;Egr2^f/f^-*lck*-Cre (Egr-DKO) (n = 3). **(C)** SAP and Bcl_xL_ expression levels in DP thymocytes from WT or dnRas mice were assessed by intracellular flow cytometry. Shown are representative histograms, and the mean and SEM of the normalized MFI of DP populations (n = 5). To normalize the MFI, we averaged the MFI for the WT mice in each experiment and considered that value 1. The bar graphs show the average and SEM of all the experiments. Significance was assessed using the unpaired t-test ***<0.001, **<0.01 *<0.05**.**

Besides the signals mediated by the TCR-CD1d interaction, development of iNKT cells requires additional signals initiated by homophilic interactions of members of the SLAM family -primarily SLAMF1 and SLAMF6 [7], and mediated by the adaptor protein SAP [14], [22]–[25]. To assess a possible role of alterations in this pathway to the phenotype observed in dnRas and Egr1,2 DKO mice, we determined whether expression of these proteins was normal in DP thymocytes using flow cytometry. As shown in Fig. 5A, the surface expression of SLAMF1, and SLAMF6 is slightly decreased in DP thymocytes from dnRas mice when compared with normal thymocytes. Other family members expressed in thymocytes such as SLAMF3 and SLAMF5 are not altered. Similarly we observed a small defect in CD1d expression in dnRas DP thymocytes. In contrast, expression of these molecules was not altered in Egr1,2 DKO DP thymocytes (Fig. 5B). These results suggest that signaling through the SLAM/SAP axis could be impaired in dnRas DP thymocytes, and this could contribute to the positive selection defect.

Since the observed defect in expression of some SLAM family members in dnRas DP thymocytes is similar to that observed in c-Myb-deficient DP thymocytes [26], and in these cells there are also defects in the expression of the signaling molecule SAP, and the survival factor Bcl_xL_, we decided to analyze expression of these molecules in dnRas mice. In contrast to c-Myb-deficient thymocytes, expression of SAP and Bcl_xL_, assessed by intracellular staining, was normal DP thymocytes from dnRas mice (Fig. 5C).

## Discussion

Although it is known that the signaling mechanisms that control iNKT cell development include a component derived from the TCR-CD1d interaction, the pathways that mediate this effect downstream the TCR are not completely understood. In this report we characterize the central contribution of the Ras/MAPK pathway to positive selection of the iNKT cell lineage, similar to its described contribution to conventional αβ T cell positive selection.

The Ras/MAPK pathway plays a central role during αβ T cells positive selection [12], [13] but it is thought to be dispensable for iNKT cell development [14]–[16]. In fact, it has been proposed that one of the roles of SLAMs in positive selection of iNKT cells is to block activation of Ras, by inducing recruitment and activation of Ras-GAP [14], [15]. The recent report that thymocytes defective in Egr-2 had a defect in generation of iNKT cells [11] made us reconsider the possible involvement of Ras in this process, since Egr-2 induction downstream the TCR requires activation of both Calcineurin and the Ras/MAPK pathway [27]. Our results clearly show that defects in the Ras/MAPK pathway result in a dramatic blockade in iNKT generation, with accumulation of immature iNKT cell precursors (CD44^-^ NK1.1^-^), suggesting a block in the early stages of positive selection. This defect seems mediated by the same downstream effectors as during conventional αβ T cell positive selection, because expression of Egr-2 and Id3 in immature iNKT cell precursors is significantly decreased. It is also possible that the defect in Ras/MAPK activation could interfere with later stages of iNKT cell differentiation that involve proliferation.

The Egr family of transcription factors plays an important role in many developmental checkpoints during T cell development. Although in some circumstances they can at least partially compensate for each other, they are not completely redundant. For example, during β-selection Egr-3 is the most important Egr factor [28], [29]. However, compound knockouts show that Egr1 also contributes in this stage [29]. Similarly, during positive selection of conventional αβ T cells, both Egr-1 and Egr-2 knockouts show a partial blockade in positive selection [20], [30]. Our results show that, contrary to previous reports [11], both Egr-1 and Egr-2 play a role during iNKT positive selection, although the effect of Egr-1 is subtle, and is only uncovered in mixed bone marrow chimeras, or in the Egr-1, Egr-2 double knockout animals, which have a much more profound block in iNKT generation than Egr-2 knockouts. It is also interesting that the defect we observe in Egr-2^f/f^ *lck-*Cre mice is less profound than the defect reported in the fetal liver chimeras from Egr-2^-/-^ mice [11]. This could be due to differences between fetal and adult hematopoiesis in the contribution of different Egr family members to the generation of iNKT cells, or to an incomplete deletion of Egr-2 in our conditional knockouts. However, we can not detect any Egr-2 message in sorted CD69^+^ thymocytes in our mice, suggesting that the deletion is very efficient. The molecular basis for the different contribution of these two closely related transcription factors to iNKT cell development remains an area of interest for future studies.

An interesting difference between the dnRas and the Egr-1, Egr-2 double knockout phenotypes is the observed small defect in the expression of SLAMF1 and SLAMF6 in DP thymocytes from dnRas mice. Given that these two members of the SLAM family are critical for the development of iNKT cells [7], it is possible that this decrease in SLAMF1 and SLAMF6 expression could also play a role in the iNKT positive selection defect, similar to what we demonstrated recently in c-Myb knockout mice [26]. However c-Myb-defective thymocytes had also a defect in the expression of SAP, the signaling molecule required downstream SLAMs [14], [22]-[25], and we did not observe any alterations in SAP expression in dnRas thymocytes. Further experiments will be required to assess the possible contribution of the SLAMF1 and SLAMF6 expression defect to the iNKT cell selection phenotype.

In conclusion, in this manuscript we present genetic evidence supporting a critical role of Ras, and its downstream effectors Egr-1 and Egr-2 for positive selection of iNKT cells, suggesting that the signaling pathways emanating from the TCR during positive selection of conventional αβ T cells and iNKT cells are similar. It will be important to characterize how this signaling component cooperates with signals derived from the SLAM/SAP axis to initiate the molecular program that characterizes this lineage, including expression of PLZF [31], [32].

## Materials and Methods

### Ethics Statement

Mice were maintained in the Oklahoma Medical Research Foundation (OMRF) AALAC accredited facility. All animals used in this study received humane care in compliance with the regulations relating to animals and all experiments involving animals were approved by the Institutional Animal Care Committee of OMRF (IACUC) (protocol #A0008).

### Mice

dnRas [12], Egr1-deficient [28] and floxed Egr2 (Egr2^f/f^) mice [19] have been described. *lck*-Cre transgenic mice were from the Jackson labs (Bar Harbor, ME). B6-LY5.2/Cr congenic mice (CD45.1) were from NCI Frederick.

### Flow cytometry

Cells were prepared, stained and analyzed as described [26]. iNKT cells were identified using murine CD1d tetramer loaded with PBS57 [33] (NIH Tetramer Facility). For isolation of iNKT HSA^hi^ and HSA^lo^ cells, total thymocytes from WT and dnRas mice were incubated with APC-labeled CD1d-PBS57 tetramer, and then magnetically enriched using anti-APC microbeads (Miltenyi Biotec Inc), as described [26]. Enriched iNKT cells were then stained with CD1d-PBS57 tetramer, anti-TcR-β, and anti-HSA, and sorted in a FACSAria (BD).

### Intracellular staining

For intracellular Bcl_xL_ staining, thymocytes were surface-stained with CD4 and CD8, then fixed with 2% paraformaldehyde (Pella) for 10 min at 37°C, and washed in P4F (PBSx1, 4% FCS, 0.1% sodium azide). Then cells were permeabilized with 0.1% Triton X-100 (Calbiochem) in PBS for 15 min at room temperature, washed twice with 1 ml P4F and stained with an Alexa Fluor® 488-conjugated anti Bcl_xL_ antibody (clone 54H6,Cell Signaling) for 1 h at RT. Cells were washed and analyzed by flow cytometry. Intracellular SAP staining was performed using BD Cytofix/Cytoperm plus kit (Becton Dickinson), according with the manufacturer's instructions. The SAP antibody was a gift from Dr. A. Veillette.

### RT-PCR

Total RNA from sorted thymocytes was extracted using Qiagen RNeasy mini kit according to the manufacturer's protocol. Reverse transcription (RT) was performed using Taqman reverse transcription reagents (Applied Biosystems). 5 ng of cDNA was used for each assay. SYBR Green real time PCR was performed using a Biorad CFX96 Real-Time PCR detection system. Gene specific forward (F) and reverse (R) primers were designed across exon-exon junction using Primer Express software (Applied Biosystems) with equivalent efficiency to the β-actin primers. Data were analyzed using the comparative CT method using CFX software (BioRad). The primers used were EGR1 Fw: 5′-AGT GGG AGC CGG CTG GAG AT-3′ RV:5′-GGG CGA TGT AGC AGG TGC GG-3′; EGR2 FW: 5′-TTG ACC AGA TGA ACG GAG TGG-3′ RV 5′- TGC CCA TGT AGG TGA AGG TCT-3′; ID3 FW 5′-GAA GCC TGA GGG CAT GGA T-3′ RV 5′-CCT CGA GGC GTT GAG TTC A-3′; β-Actin FW 5′-CAA CGA GCG GTT CCG ATG -3′ RV 5′-GCC ACA GGA TTC CAT ACC CA-3′.

### Retroviral transduction of hematopoietic progenitors

Preparation of viral supernatant, purification and infection of Lin^-^ progenitors was performed as described [20] [34].

### Generation of mixed bone marrow chimeras

Bone marrow cells were harvested from the femurs and tibiae of F1 (C57BL/6 x B6-Ly5.2) (CD45.1/CD45.2), and Egr-1^-/-^ (CD45.2). B6-LY5.2/Cr (CD45.1) recipient mice were lethally irradiated with 1300 rad from a cesium source. A 1∶1 mixture of F1(CD45.1/CD45.2) and Egr-1^-/-^ (total of 1×10^6^ cell) bone marrow cells were retro-orbitally injected into the hosts. Animals were given antibiotic-containing water and housed in sterile microisolator cages. Mice were analyzed eight to twelve weeks after reconstitution.

### Statistical Methods

Normal distribution of the data was assessed using the Kolmogorov-Smirnov test. Depending on the results, statistical significance was determined by the Student's *t* test (for parametric data) or by the Mann-Whitney test (for non-parametric data).
